# Building a Tailored, Patient-Guided, Web-Based Self-Management Intervention ‘ReumaUitgedaagd!’ for Adults With a Rheumatic Disease: Results of a Usability Study and Design for a Randomized Control Trail

**DOI:** 10.2196/resprot.5735

**Published:** 2016-06-23

**Authors:** Judy W Ammerlaan, Olga K Mulder, Nienke C de Boer-Nijhof, Bertha Maat, Aike A Kruize, Jaap van Laar, Harmieke van Os-Medendorp, Rinie Geenen

**Affiliations:** ^1^ University Medical Center Utrecht Department Rheumatology and Clinical Immunology Utrecht Netherlands; ^2^ Dutch Arthritis Foundation Amsterdam Netherlands; ^3^ University Medical Center Utrecht Department Dermatology and Allergology Utrecht Netherlands; ^4^ Utrecht University Department Clinical and Health Psychology Utrecht Netherlands

**Keywords:** Web-based, self-management, tailored, intervention, pilot study, randomized controlled trial, personal goal, rheumatic diseases

## Abstract

**Background:**

The chronic nature of rheumatic diseases imposes daily challenges upon those affected and causes patients to make daily decisions about the way they self-manage their illness. Although there is attention to self-management and evidence for the desirability of tailored interventions to support people with a rheumatic disease, interventions based on individual needs and preferences are scarce.

**Objective:**

To provide a systematic and comprehensive description of the theoretical considerations for building a Web-based, expert, patient-guided, and tailored intervention for adult patients with a rheumatic disease. Also, to present the results of a usability study on the feasibility of this intervention, and its study design in order to measure the effectiveness.

**Methods:**

To fit the intervention closely to the autonomy, needs, and preferences of the individual patient, a research team comprising patient representatives, health professionals, Web technicians, and communication experts was formed. The research team followed the new guidance by the Medical Research Council (MRC) for developing and evaluating complex interventions as a guide for the design of the intervention.

**Results:**

Considerations from self-determination theory and a comprehensive assessment of preferences and needs in patients with a rheumatic disease guided the development of the Web-based intervention. The usability study showed that the intervention was useful, easy to use, and accepted and appreciated by the target group of patients. The planned randomized controlled trial is designed to be conducted among 120 adults with a rheumatic disease, who are assigned to the self-management intervention or a self-help control group. Both groups will be asked to formulate personal goals they want to achieve concerning their self-management. Progress toward the personal goal is the primary outcome measure of this study. Self-reported Web-based measures will be assessed before randomization at baseline, and 3 and 6 months after randomization. Also, feasibility and adherence to the Web-based self-management intervention as process outcomes will be evaluated.

**Conclusion:**

By identifying the individual goals at the beginning of the intervention and customizing the intervention to the individual patient, we aim to improve the usefulness and effectiveness of the Web-based self-management intervention. If proven effective, ReumaUitgedaagd! Online will be implemented in the Netherlands.

## Introduction

### Background

Having a rheumatic disease often leads to symptoms of pain, fatigue, and physical constraints that are part of a reduced health-related quality of life [1]. The chronic nature of this disease imposes daily challenges upon those affected and causes patients to make daily decisions about the way they manage their illness [1-3]. The question is not “whether” patients self-manage their (chronic) illness, but “how” they do this [4]. The ‘(individual’s) ability to manage the symptoms, treatment, physical, and psychosocial consequences and life style changes inherent in living with a chronic condition’ has been defined as self-management [5]. Interventions to improve self-management commonly combine information-based and cognitive-behavioral strategies [6]. In the last decade, several interventions have been developed to improve self-management. For people with a rheumatic disease, the Arthritis Self-Management intervention (ASMP) of Stanford University is the most recognized and studied self-management intervention [7]. The ASMP intervention, based on the self-efficacy theory of Bandura [8] is led by expert patients and is designed to help people with arthritis gain confidence in their ability to control their symptoms and the impact of their condition on their lives [7,9,10]. For patients with rheumatoid arthritis and osteoarthritis, participating in an ASMP led to improved health behavior (cognitive symptom management, communication with physicians, dietary habit, exercises, and relaxation) and a decrease of depression. However, decreases in fatigue and anxiety were found not to be significant [6,7].

With the growing opportunities and use of the Internet, a Web-based self-management version of the ASMP intervention for patients with long-term conditions was developed in 2007 [11]. Evaluation of the effectiveness of this intervention after 12 months showed significant improvements on health status measures like distress, pain, and self-efficacy. In 2011, based on the ASMP intervention and the self-efficacy theory of Bandura and in cooperation with the Dutch Arthritis Foundation and young adults from the transition outpatient clinic of University Medical Center Utrecht, we developed a Web-based self-management intervention for young adults up to the age of 25 years [12]. The aim of that intervention was to enhance young adults’ self-management in coping with their rheumatic disease.

With the expansion of the Web-based intervention in the Netherlands, older adults with rheumatic diseases also expressed their need for a Web-based self-management intervention. In order to meet this need, the Dutch Arthritis Foundation gave us a grant to develop a Web-based intervention for adults from the age of 25 years and older. The goal of this research protocol was to describe (1) the theoretical considerations that guided the development of this Web-based intervention for adult patients with rheumatic diseases, (2) the contents of the intervention, (3) the results of a pilot study to study the usability of the intervention, and (4) the study design in order to examine the effectiveness of the intervention.

### Toward a Patient-Guided Intervention

As we inferred from our experiences with the development and research pertaining to the Web-based intervention for the young adult group, collaboration with the end-users in all phases of development of a Web-based self-management intervention is crucial and influences the actual use, adherence, and effectiveness [13]. Based on this notion and to fit the intervention closely to the autonomy, needs, and preferences of the patients, we formed a research team consisting of patient representatives of different ages, health professionals, Web technicians, and communication experts. The research team followed the new guidance for developing and evaluating complex interventions by the Medical Research Council (MRC) [14] as a guide for the design of the intervention. The guidance included the following phases: development, feasibility and piloting, and evaluation and implementation. During the development phase, the aims were to determine a theoretical foundation and to develop the structure and content of the first draft. To achieve these aims, we first screened the scientific literature for theoretical considerations and effectiveness of (Web-based) self-management interventions for people with a chronic or rheumatic disease. The search for qualitative and quantitative articles was conducted in Medline, the Cumulative Index to Nursing and Allied Health Literature, Web of science, PsycINFO, and Pubmed. We searched for studies published in English, which used the words: “self-management,” “chronic disease,” “rheumatic disease,” “adults,” “theoretical foundations/considerations,” and “effectiveness” in different combinations. No publication year limit was used. Secondly, a focus group and concept mapping study was performed to assess preferences and needs of adult patients with a rheumatic disease regarding the structure and content of the future Web-based self-management intervention.

#### Theoretical Considerations

Although there is growing attention for interventions that are customized to individual patients with chronic diseases, the structure and contents are generally still protocol-based on group preferences [6,15]. And, disappointingly, to date there is no consistent (long-term) evidence of the efficacy of self-management interventions for patients with a chronic disease in general [6,16]. This might be due to various reasons, including diversity of interventions, insufficient theoretical foundation, and the heterogeneity of the patient populations [16]. Moreover, positive mean group outcomes may disguise that a substantial proportion of patients did not comply with or respond to the intervention [6,16]. A basic assumption about self-management is that when the intervention is customized to the individual needs and situation of the patient, the patient will be more motivated, adhere better, and benefit more and for a longer time [6,15,16]. Thereby, a change in behavior and long-term adherence to changed behavior is expected to be greater when a patient experiences a meaningful rationale for change, values the change in behavior positively, and aligns it with other central values and lifestyle patterns [2,3,17]. Consistent with these assumptions is Self-determination Theory of Ryan and Deci [17], which emphasizes the importance of keeping goals of behavior change (like improvement of self-management) close to the autonomous motivation of people. In this theory, three basic needs determine motivation: autonomy, competence, and social relatedness [17]. Among these three, autonomy is considered as the most central need: if a behavior is autonomous, it is voluntary, originating from one’s own values and self-determination. Competence refers to the necessity to experience that one is really able to achieve something, and is related to the construct of self-efficacy [10]. The third basic need, social relatedness, is the extent to which one finds support in one’s environment, including support from a trainer or professional. High levels of autonomy, competence, and social relatedness enhance self-regulation.

### Needs Assessment

An important part of the development phase consisted of a needs assessment, conducted by the combination of a focus group and concept mapping design (J.W. Ammerlaan, et al, unpublished data, 2016). Online focus group interviews among adult patients with rheumatic diseases in the Netherlands, a card sorting task, and hierarchical cluster analysis yielded an extensive overview of the individual preferences regarding structure and content. Patients preferred an intervention tailored to their needs, stage of life, and goals. Also, an expert patient as a trainer, the opportunity to be in contact and to share with others, and the ability to follow the intervention at one’s own pace were preferred. With respect to needs for content of the intervention, hierarchical cluster analysis yielded 11 clusters involving increasing individual knowledge of treatment and consequences for daily life, skills including managing emotions, managing, the fluctuations of disease, and dealing with health professionals and social authorities. Self-regulating their own lives, including requesting support from their spouse, family, or coworkers, setting boundaries and the ability to communicate adequately, and dealing with pregnancy or intimacy issues and taking care of kids. Based on the data from this needs assessment and the theoretical considerations, the first draft of the Web-based self-management intervention (in Dutch: ReumaUitgedaagd! Online) was developed.

## Methods

### Design of the Web-Based Intervention

ReumaUitgedaagd! Online is a Web-based, password protected, tailored, self- management intervention for adults with a rheumatic disease, aimed at enhancing patients’ self-management skills. The participants perform the intervention individually, are coached by a trainer, and have online contact with other participants on a discussion board. The role of the trainer is to support participants during the Web-based intervention in becoming a good self-manager and achieving their personal goals. The trainers are adults who also have a rheumatic disease. They are recruited through the website of the Dutch Arthritis Foundation and selected through assessments and interviews conducted by a professional coaching organization (Work21), in close cooperation with the Dutch Arthritis Foundation and the University Medical Center Utrecht. The selection process used questions about motivation, perceptions of self-management, the self-determination theory and strategies derived from the theoretical foundation, and goals of the Web-based intervention to identify those trainers who could adhere to the basic tenets of the intervention. Finally, the expert trainer was trained through a 3-day train-the-trainer (TTT) educational intervention. The TTT intervention consisted of following the intervention as a participant, knowledge of different themes, and teaching Web-based training skills. The trainers are given a volunteer contract and receive a stipend from the Dutch Arthritis Foundation. The basic needs of autonomy, competence, and social relatedness, derived from Self-Determination theory, are embedded in the intervention and combined with elements of skills training and modelling, based on the Self-efficacy theory [8]. Autonomy is taken into account by customizing the intervention to three individual needs and goals, which the participants choose at the beginning of the intervention. The participants choose thematic modules based on these individual needs and goals. For example: a woman who wants to learn more about coping with the consequences of her disease at work, may choose the ‘Work’ module, while a man who wants to increase his physical fitness may choose the ‘Exercises’ module. Competence is increased by making action plans, reflecting on one’s own behavior by performing exercises, or sharing the output of exercises on the discussion board to receive feedback or support from other participants and the expert trainer. Social relatedness is achieved through the support of the expert trainer via individual chats and the message box, and also by sharing experiences and giving feedback and support with other participants.

### Content of the Web-Based Intervention

The Web-based self-management intervention consists of four components: nine thematic modules (willing, knowing, skills, feeling, living together, influence, exercise, work, and moving on), a chat application, a discussion board, and a message box.

#### Modules

**Figure 1:**
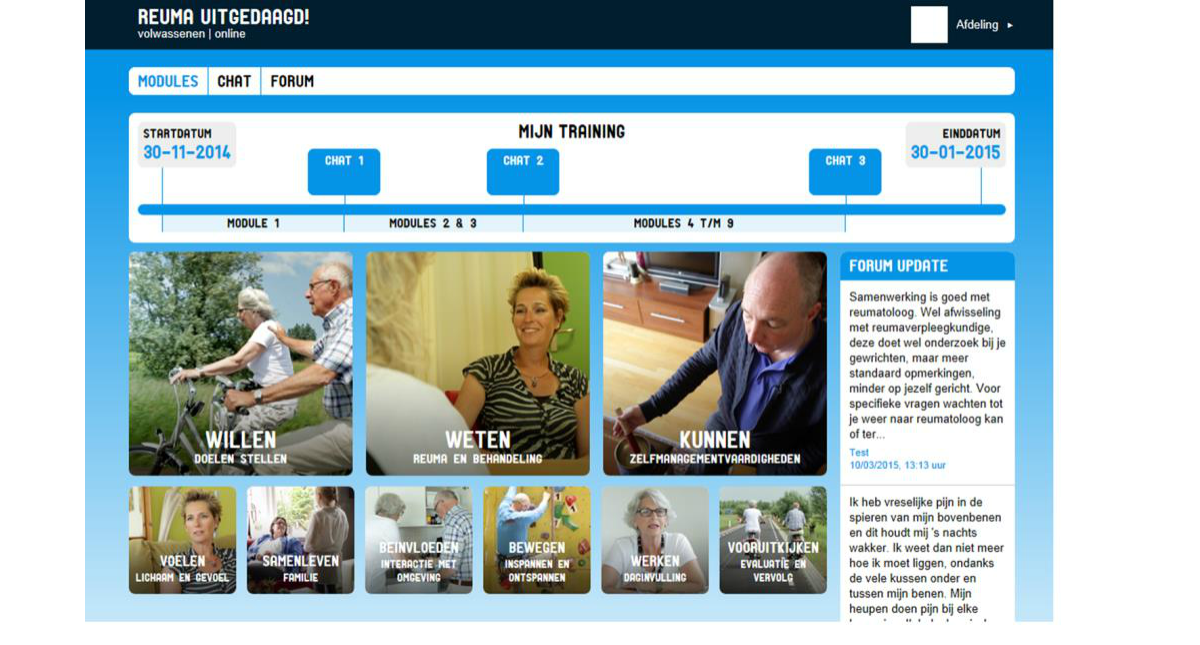
Screen of the homepage, showing the nine modules of the online Reuma Uitgedaagd! (in Dutch).

Each module involves a specific theme. Both informative text about the theme and exercises are included. The information and the exercises are supported by short videos in which people with a rheumatic disease or a member of the multidisciplinary team tell about their experiences with arthritis. The content of the modules is described in Table 1. The participant performs the intervention individually and has 2 months to complete it. The total time investment for the participants is between 4 and 9 hours (approximately 30-60 minutes per module). The first three modules and the last module (‘willing’, ‘knowing’, ‘skills’, and ‘moving on’) are mandatory for all participants. The participant can choose other modules depending on his or hers personal goals. The nine modules are displayed in Figure 1.

#### Chat Sessions

The intervention includes three chat sessions between the participant with the trainer (after finishing module 1, after finishing module 3, and after finishing module 9). During the chat sessions, the trainer discusses the progress of the intervention and answers questions from participants. The duration of a chat session is approximately 15 to 30 minutes. The participant also has the ability to individually contact the trainer via a message box.

#### Discussion Board

The purpose of the discussion board is to exchange experiences between participants and trainers. In some exercises the participants put their output on the discussion board to start a discussion. For instance, they report how they tend to deal with being dispirited and whether or not they feel the strategy is proving to be successful.

#### ‘Look and Feel’ of the Intervention

Based on the preferences of the research team, the design of the Web-based intervention was made attractive by using ‘colorful, real-life pictures of people of different ages’ to support information and exercises. Secondly, pictures of people, performing activities based on the content of the module, were used as pictograms to navigate. Thirdly, the videos to support the informative text of the modules were directed and produced by a professional company. Finally, a voice over was added to assist visually impaired participants.

**Table 1:** Content of the Nine Modules of the Web-Based Self-Management Intervention and Exercises

Module	Contents	Exercises
**1. Willing (formulating personal goals)**	Self-management	Awareness of self-management
	Priorities in life (getting to know yourself)	Evaluating self-management
	Setting and achieving personal goals	Life values (priorities in life)
		Formulating personal goals for the training
**2. Knowing (disease-specific information and treatment)**	A rheumatic disease: what does that mean?	Knowledge Quiz: what do you (already) know of your disease?
	Treatment possibilities	Gaining insight into treatment and treatment goals
	Getting control over one’s disease and treatment	Working together with your physician and health professional
		Pain and fatigue diary
**Additional**	Medication	Practice and evaluation of consultation in the hospital
	A consultation in the hospital: how do you prepare yourself?	
**3. Skills (self-management skills)**	Being in charge:making choices	Evaluating your own behavior: making choices
	Problem solving	Circle of influence and engagement
	Communication	Feedback in your daily life
	To give and receive feedback	Saying no
	Setting boundaries	Recognizing your own coping scale
	Coping: dealing with consequences	
**Additional**		Self-assertiveness test
**4. Feeling (body, mind, and emotions)**	Having a rheumatic disease; what’s next?	Loss of health; what does that mean to you?
	Consequences of having a rheumatic disease on your body, your mind, and socially	Feeling blue
	Pain, fatigue, and negative emotions	Evaluation of a situation to get insight into the influence of one’s thoughts, behavior, and feelings
	Your own influence	Evaluation of the pain and fatigue diaries
**Additional**	Processing phases in the loss of health	Dealing with the loss of health
	Tips for handling pain	Relaxation exercises
	Tips for handling fatigue	To puzzle over: what can you do?
	To rack one’s brain: what can one do about it?	
**5. Living together (family and spouses)**	Communicating with family and friends	Relationships
	Kids and stuff	Intimacy
		Sexuality
		Asking for help from your representatives or friends
**Additional**	Getting pregnant and having kids	Communicating with your partner
	Taking care of kids	
	Communicating with one’s children	
**6. Influence (interaction with your environment)**	How to influence one’s environment?	Explain your disease and consequences
	Dealing with lack of understanding (invalidation)	Asking for help: sharing experiences
	Asking for help	
**7. Exercise (sport, exert, and relaxation)**	Exercise and having a rheumatic disease	Your exercises
	Motion and physical activity	Exercise diary
	Pain and overload	Action plan
	Exertion and relaxation	Relaxation
**Additional**	Exercise and different rheumatic diseases	
**8. Work (daily activities)**	Suitable work	What’s a suitable job for you?
	Dealing with invalidation at work	Who knows that you have a rheumatic disease at work?
	Dealing with fatigue and stress at work	Dealing with obstacles
	Rights and obligations	
	Going to school or university	
	To apply for a job	
**Additional**	Preparing for an interview with your colleagues or boss	Preparing for an interview with your colleagues or boss
	Being sick and getting back to work/school	
	Work adaptions	
**9. Moving on (evaluation and looking forward)**	Your personal goals	Self-management: reflection of your own knowledge and skills
	Action plan for the future	Action plan for the future
	Evaluation	Evaluating your own goals
**Additional**	An example of an action plan	

### Usability Testing

#### Design

The first draft of the Web-based self-management intervention was tested in a quantitative pilot study, using the three concepts of the Technology Acceptance Model (TAM) [18]: perceived usefulness, perceived ease of use, and intention to use. According to TAM, the usability of a particular technical innovation can best be predicted by an individual’s intention to use or re-use the innovation. This intention is determined by two components: (1) perceived ease of use, which can be defined as “the degree of ease, associated with the use of the applications,” and (2) perceived usefulness, which can be defined as “the degree to which an individual believes that using applications will help him to attain gains or to increase personal performance.” [18].

The participants of the pilot study were given 3-weeks’ access to the Web-based self-management intervention to examine and apply the contents of the intervention. After 3 weeks, the participants completed a Web-based questionnaire on usability (based on the TAM).

#### Population

Adult patients with access to a computer with Internet, sufficient Internet skills, diagnosed with a rheumatic disease, and being able to read and write in Dutch were included. Participants were recruited through websites, Facebook, and Twitter accounts of the Dutch Arthritis Foundation [19] and ReumaUitgedaagd! [20]. All patients gave informed consent via the Internet.

#### Variables and Outcome Measures

Demographic variables like age and type of rheumatic disease and self-reported Internet-skills (measured on a 5-point Likert scale from very bad to very good) were collected to describe the group. Usability as primary outcome measure was operationalized using the three concepts of the TAM with 11 questions on a 5-point Likert scale (from totally disagree to totally agree) with the possibility to give additional comments. One question on ‘overall satisfaction’ was added, using a numeric rating system (NRS) from 0 (not satisfied) to 10 (most satisfied) (see Textbox 1).

**Questions to Measure the Three Concepts of the Technology Acceptance Model on Usability (All questions start with: “Now that you have seen the Web-based intervention…”)**

Perceived usefulness

- Did you perceive the content of the intervention to be useful?
- Did you perceive the content of the intervention as understandable?
- Did you perceive the exercises in the intervention to be useful?
- Did you perceive the content of the exercises as understandable?
- Did you perceive the intervention to be useful as a supplement to usual health care?
- Did you perceive the intervention to be useful in dealing with the consequences of having a rheumatic disease in daily life?

Perceived ease of use

- Did you perceive the Web-based self-management intervention to be easy to navigate?
- Could you easily find what you were looking for?

Intention to use

- Would you participate again, knowing now the content and structure?
- Would you recommend the Web-based self-management intervention to others (knowing now the content and structure)?

Overall satisfaction

- How do you rate your overall satisfaction with the intervention?
- How do you rate the look and feel of the intervention?

#### Results

Twenty-three respondents (22 women, mean age of 47 years) were given access to the Web-based intervention to test the usability. Most of them were diagnosed with inflammatory arthritis (16/23, 70%). Other diagnoses were osteoarthritis and fibromyalgia. Ninety-one percent (21/23) of participants rated their Internet-skills as ‘very good’. Two participants rated their skills as average.

Ninety-one percent (21/23) of the participants indicated the content and exercises as easy to understand and useful (ie, agree/totally agree on the Likert-scale). The majority of the participants (21/23, 91%) indicated the intervention to be useful in dealing with the consequences of having a rheumatic disease in daily life. The navigation on the site itself was rated somewhat lower with 70% (16/ 23) of participants being critical about the menu with thematic modules on the homepage and finding their way on the website. The look and feel of the intervention was recognized by 78% (18/23) of participants as pleasant.

In terms of intention to use: 78% (18/23) would participate in the Web-based intervention themselves and 91% (21/23) would recommend it to others. The mean satisfaction score of the Web-based intervention was rated 7.9 (range 4-10) on a scale of 0 (not satisfied) to 10 (most satisfied).

#### Conclusion

Considering the three concepts, we concluded that the Web-based intervention was to be recognized as being useful and easy to use. Participants stated that they were likely to participate; now they were familiar with the content and structure. To improve the navigation and menu of the intervention, numbers were added to each module in order to indicate the sequence of the modules **.**

### Study Design in Order to Measure the Effectiveness of the Newly Developed Web-Based Intervention

#### Design

To evaluate the Web-based self-management intervention, we have planned a randomized controlled trial with an intervention and a self-help control group and a 6-month follow-up period among adults in the Netherlands having a rheumatic disease. The control group will be put on a waiting list and will cross-over to the intervention after 6 months. Participants in the intervention group will be given access to the Web-based self-management intervention ReumaUitgedaagd! Both groups will receive usual care, based on the medical standard guidelines of the Dutch Association of Rheumatology [21], which also includes attention for self-management by the use of informational and educational materials that are normally used by patients to promote self-management. These materials are offered on the website of the Dutch Arthritis Foundation. Measuring the effectiveness means that we investigate whether there is an additional effect of the Web-based intervention in the intervention group on top of the care that is usually offered. The medical-ethical review board of the University Medical Center Utrecht in the Netherlands has approved the design and the procedures of this study.

#### Participants

Because we already have a Web-based self-management intervention for young adults (from 16-25 years), adults ≥26 years, having a rheumatic disease, diagnosed at least 2 years before inclusion by a rheumatologist or a General Practitioner, are eligible for this study. In addition to having an Internet connection, patients need to have proficiency in the Dutch language and not having previously participated in a self-management intervention. Having a psychiatric disorder or being under (recent) treatment by a psychologist or psychiatrist are criteria for exclusion from this study. The participants will be recruited via the Internet through websites, Facebook, and Twitter accounts of the Dutch Arthritis Foundation [19] and ReumaUitgedaagd! [20]. After having signed consent forms, they receive information about the study and an information paper on goal setting with instruction and some examples of goals derived from the study on needs (J.W. Ammerlaan, et al, unpublished data 2016). After 1 week, a telephone call will be set up between the researcher and the participant to check the inclusion criteria and to answer any questions about the study. The participants will also be asked to think about three individual goals concerning their self-management they want to achieve. One week later, a second phone call will take place between the researcher and the participant to set the final three individual goals and to inform the participant about the randomization procedure. To warrant objectivity and standardization as much as possible, standardized scripts will be used for the two contacts. The telephone calls are conducted by an independent interviewer (OM) who is not involved in the care of patients with a rheumatic disease.

#### Randomization

Randomization will take place after informed consent and completion of the goal-setting procedure, using a computerized application of the University Medical Center Utrecht. This is an automated process with no interference from the investigators. We will use a stratified block randomization to decrease the likelihood of imbalance between three conditions (arthritis, osteoarthritis, and soft-tissue rheumatism). After randomization, the participants will be informed by the researcher if they are assigned to the intervention or control group. The participants of the intervention group will then start with the Web-based self-management intervention and be asked to work through the intervention within 2 months.

#### Outcome Measurements

**Figure 2:**
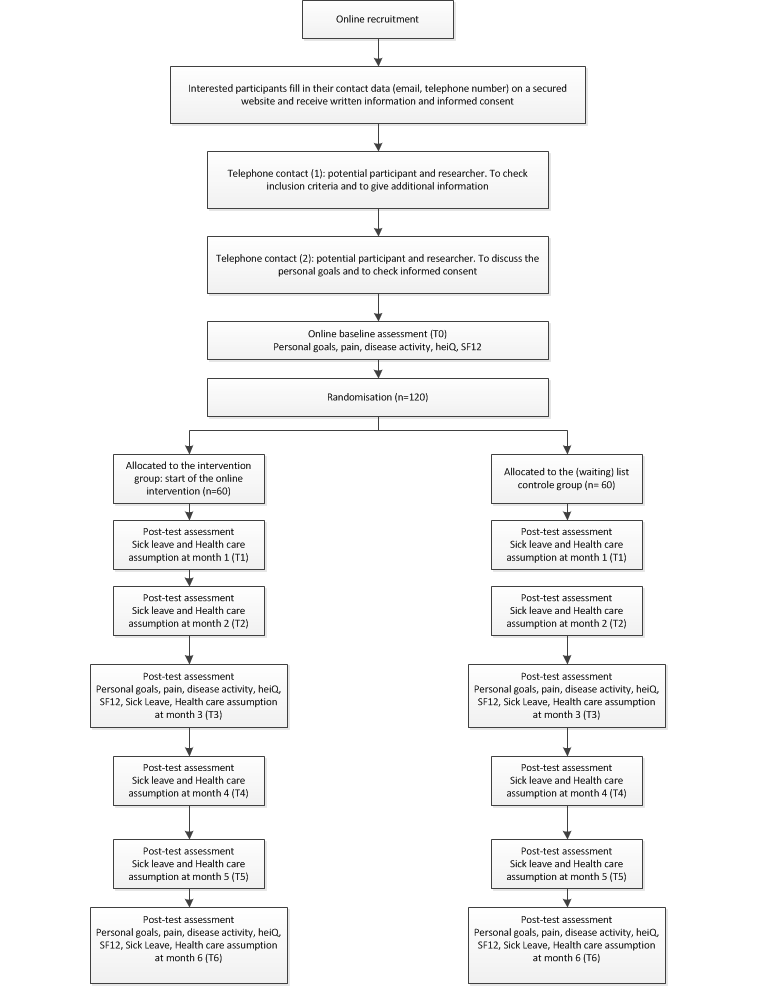
Time frame and flow-chard study design.

In this study individual outcome measures, generic outcome measures, and process outcomes measures will be collected, most of them via the Internet with questionnaires, self-reported by the participants. Demographic variables including age, sex, disease duration, diagnosis, marital status, current treatment, education level, work, and comorbidity at baseline will be collected in order to characterize the group of participants. The timeframe for collecting the outcome measures is displayed in Figure 2.

##### Individual Outcomes: Goal Accomplishment

A crucial and novel aspect of this study is that the intervention is customized to the needs of the participant. This is one of the reasons why the change –progress toward– the main personal goal is chosen as the primary individual outcome measure. Studies show that on individual outcome measures the effect is larger than on generic outcome measures [22-24], which is not unexpected because many of the patients are already on a functional level of generic outcome measures; even more so, given that patients with psychopathology are excluded. The three personal goals that the patient wants to accomplish are in a telephone interview with the researcher checked according to the following criteria: (1) the individual goals are aligned to the content and overall aim of the self-management intervention, (2) the goals focus on ‘knowledge’ and ‘skills’, and (3) the participant feels that each goal is achievable. In addition, the participant is asked to prioritize these goals (the first goal being the most important goal). Progress on the highest priority personal goal is the primary outcome of the study. Evaluations of the second and third personal goals are also conducted as (secondary) individual outcome measures.

The personal goals are measured with a Web-based NRS. The participant is asked to indicate with a score from 0 to 10 on the NRS to what extent he or she achieved this goal. The content of the primary goal can differ per person but the rate of change can be compared between subjects because they are measured on the same scale.

##### Generic Outcomes

The following generic outcome measures are assessed (all self-reports): pain, disease activity, self-management skills, quality of life, and sick leave.

Self-reported pain and disease activity will be scored by the participant on a Web-based NRS from 0 to 10. The higher the score, the more pain or disease activity.

Self-management will be measured with the Dutch translation of the Web-based Health Education Impact Questionnaire (heiQ) [25], which consists of 40 questions with scores ranging from 1 (not at all true) to 4 (exactly true) and are organized into a set of eight scales: health-directed behavior, positive and active engagement in life, emotional well-being, self-monitoring and insight, constructive attitudes and approaches, skills and technique acquisition, social integration and support, and health navigation. In a recent study of patients with a chronic disease, the heiQ scales showed good internal consistency, with Cronbach’s alpha ranging from 0.70 to 0.89 on the eight independent scales, and high construct validity [26].

Quality of life will be assessed with the Web-based Medical Outcomes Study 12-item Short Form Health Survey (SF-12) [27], which includes eight questions on functional status, three questions on general well-being, and one question on general health. The psychometric properties of the SF-12 are good [27].

Sick leave is measured with three questions regarding (1) working in a paid job (yes/no/how many days a week), (2) sick leave during the past month, and (3) reasons for sick leave. Two measures of health care assumption are recorded as follows: self-reported visits to general practice, medical specialist, or physiotherapist, and whether or by whom support is offered to achieve the personal goals.

##### Process Outcomes

Feasibility is measured as a process outcome in the effectiveness study to evaluate the intervention in real-life in a larger group. Feasibility will be measured within the intervention group using the three concepts of the TAM [18,28] (see Textbox 1).

Use and adherence of the Web-based self-management intervention are digitally measured by Google Analytics within the intervention group. This was done by counting: (1) the number of starting and finishing participants within the time period, (2) the number of started and finished exercises, (3) the number of logins, (4) the number of messages that were put on the discussion board, (5) the number of contact moments with the expert trainers, and (6) the number of messages on the message box.

#### Power Calculation

To be able to compare our results with previous evaluations of self-management interventions, power calculation was based on the generic outcome parameters. In previous research, the generic measures of self-efficacy (which is close to our measurement of self-management skills) and functioning (which is part of our quality of life measurement), small to moderate effect-sizes (d) were found varying from 0.21 to 0.42 [11,29,30]. An effect-size d of 0.30 is similar to an effect-size f of 0.15 in repeated measures analysis of variance. In the current study, to be able to find a small to moderate difference (f=0.15) between the experimental and control groups using repeated measures analysis of variance, the total sample size needs to be N=90 (2 groups of n=45): G*Power3: f=0.15, 1-ß=.80, α=.05 two-tailed, r=.50, two groups, two repeated measures (baseline vs one post-therapy measurement) [31]. Taking a dropout rate of 25% into account, we decided to recruit 120 participants. The expectation of a small to moderate effect-size on these generic outcome measures may be explained because patients already have reasonable scores on self-management and quality of life at the start of the study. And there is little reason to expect that the intervention will affect other generic measures such as disease activity and sick leave.

As the crucial aspect of the current study is that the intervention is customized to the needs of the individual participant, the change on the main individual goal is chosen as primary outcome measure, and the change on the other two individual outcome measures (evaluation of the second and third personal goal) is considered important as well. Our sample size is large enough to examine differences in this primary outcome measure. Based on previous studies with individual outcome measures [22-24], we expect a moderate effect-size (d=0.5) for the control group and a large effect-size (d=1.2) for the intervention group resulting in a moderate to large (d=0.7, f=0.35) effect-size when comparing the intervention group and the control group using the three individual primary and secondary outcome measures. The calculated sample size is therefore considered to be safely chosen to test the main individual goal and both secondary individual outcome measures.

#### Statistical Analysis

Demographic and disease-specific outcomes will be descriptively presented per group, where possible, with means and standard deviations. The Consolidated Standards of Reporting Trials statement [32] will be used to report the results of this study. Quantitative data will be entered into a SPSS database. Effect analyses will be done according to intention to treat analysis by means of linear mixed-models for longitudinal measurements with random intercept. Fixed effects for group, time, and group × time will be included in the model. Sick leave and health care use will be counted and differences between both groups will be analyzed using parametric tests or nonparametric tests, depending on the distribution of the data. Process outcomes, feasibility, use, and adherence will be analyzed with descriptive statistics.

## Results

Patient inclusion and data collection will be completed in February 2017.

## Discussion

### Implications of the Intervention

A comprehensive assessment of the preferences and needs of patients with a rheumatic disease was used to build ReumaUitgedaagd! Online, guided by Self-Determination theory [17]. The usability study showed that the intervention was considered useful, easy to use, and accepted and appreciated by the target group of patients. These results predict that the intervention will be used to improve the use and effectiveness of this intervention, individual goals based on personal needs are identified at the beginning of the intervention and the intervention is customized to the individual patient. Because the intervention is personalized and guided by needs and preferences of patients, a low drop-out rate is expected.

According to the MRC framework [14], which was used by the research team to develop and evaluate the Web-based self-management intervention, this intervention can be defined as complex, taking into consideration the components, the required behaviors, and level of difficulty for both participants and trainer. The intervention is also flexible and customized to the individual participant. Although the MRC framework does not recommend active involvement of the users in the development or evaluation of the intervention, the knowledge and experiences of patient representatives were embedded in all phases of the framework. The aim of involving the users was to the use, acceptance, adherence, and effectiveness of the intervention [13,18]. Although we conducted a usability study in an earlier phase of the MRC model, we will measure additional process outcomes like feasibility, use, and adherence to gain knowledge of the working elements of the newly developed Web-based intervention.

### Conclusion

Strong features of this Web-based intervention are that it is guided by needs and preferences of patients, that the precise contents of the interventions are customized to the individual patient, and that also the outcome measures fit the self-management goals that are really important to the individual patient. This makes the intervention an example of personalized, patient-centered care. If proven effective, ReumaUitgedaagd! Online will be implemented in the Netherlands.

## Abbreviations

ASMP: arthritis self-management intervention
heiQ: health education impact questionnaire
MRC: medical research council
NRS: numeric rating scale
SF-12: short form health survey
TAM: technology acceptance model
TTT: train-the-trainer
